# HPV Infection and Cervical Screening in Socially Isolated Indigenous Women Inhabitants of the Amazonian Rainforest

**DOI:** 10.1371/journal.pone.0133635

**Published:** 2015-07-24

**Authors:** Allex Jardim Fonseca, Daniela Taeko, Thiciane Araújo Chaves, Lucia Dayanny da Costa Amorim, Raisa Saron Wanderley Murari, Angélica Espinosa Miranda, Zigui Chen, Robert David Burk, Luiz Carlos Lima Ferreira

**Affiliations:** 1 Postgraduate Program in Tropical Medicine, Tropical Medicine Foundation Dr. Heitor Vieira Dourado, Universidade do Estado do Amazonas, Manaus, Amazonas, Brazil; 2 Department of Health Sciences Research, Universidade Federal de Roraima, Boa Vista, Brazil; 3 Center for Infectious Diseases, Universidade Federal do Espírito Santo, Maruípe, Vitoria, Brazil; 4 Department of Pediatrics, Albert Einstein College of Medicine, New York, United States of America; 5 Department of Microbiology & Immunology, Albert Einstein College of Medicine, New York, United States of America; 6 Department of Epidemiology & Population Health, Albert Einstein College of Medicine, New York, United States of America; 7 Department of Obstetrics, Gynecology & Women’s Health, Albert Einstein College of Medicine, New York, United States of America; 8 Department of Tropical Pathology, Tropical Medicine Foundation Dr. Heitor Vieira Dourado, Universidade do Estado do Amazonas, Manaus, Amazonas, Brazil; Istituto Nazionale Tumori, ITALY

## Abstract

**Objective:**

Indigenous women from the Amazon regions have some of the highest rates of cervical cancer in the world. This study evaluated cervical cytology and human papillomavirus (HPV) in native women that differ by lifestyle and interaction with western society. Yanomami women are isolated deep in the Amazon with a hunter/gatherer lifestyle. Macuxi and Wapishana women live in proximity to western society.

**Methods:**

To select a representative group of women from each district, random cluster sampling was used, considering each registered village as a cluster. Cervical samples were collected for cytology and HPV detection and typing by PCR amplification and next generation sequencing. The study was approved by the National IRB and by tribal leaders.

**Results:**

664 native women were enrolled from 13 indigenous villages (76% participation rate). Yanomami women had higher rates of abnormal cytology (5.1% vs. 1.8%, p = 0.04) and prevalent HR-HPV (34.1% vs. 19.2%, p<0.001). Yanomami women >35y of age were significantly more likely to have HR-HPV, whereas women ≤35y did not significantly differ between groups. Prevalence of HPV was significantly different amongst geographically clustered Yanomami women (p<0.004). The most prevalent HPV types in the entire group were HPV31 (8.7%), HPV16 (5.9%) and HPV18 (4.4%).

**Conclusion:**

Isolated endogenous Yanomami women were more likely to be HPV+ and rates increased with age. Study of HPV in isolated hunter-gather peoples suggests that long-term persistence is a characteristic of prehistoric humans and patterns reflecting decreased prevalence with age in western society represents recent change. These studies have implications for cervical cancer prevention and viral-host relationships.

## Introduction

Among the nearly 500,000 new cases of cervical cancer (CC) estimated annually worldwide, 80% occur in middle or low income countries such as Brazil[1], where 15,900 new cases of cervical cancer occur each year (19/100,000)[2]. In the Brazilian Amazon region, CC is a major public health problem. A population-based study[3] conducted in 2010 revealed an annual incidence rate of 46/100.000 in the Amazonian region, which is the highest incidence rate in Brazil. Demographicaly approximately 15% of the Brazilian Amazonian population is constituted by indigenous peoples[4]. However, 23% of cervical cancer cases in this region are reported in native women[3], suggesting a higher risk of CC (estimate incidence rate 110/100,000) which is amongst the highest rates reported in the world[5].

Despite improving the health of indigenous populations being identified as a priority for Healthcare Organizations[6], the health and epidemiological profile of indigenous women remains unknown, due, in part, to the lack of research and the precariousness of information systems on morbidity and mortality in these groups[7]. In South America, few studies have assessed the prevalence of HPV and cervical intraepithelial lesions in indigenous women in the jungle, whereas many studies have investigated indigenous groups from the most urbanized regions of the continent[8–10]. In indigenous groups in South America with permanent interactions with western society, the reported prevalence of HPV in asymptomatic women ranged from 14 to 60%. A study that analyzed the HPV16 variants in Quechua indigenous women (an ethnic group integrated within the surrounding society) that inhabit the border region between Argentina and Brazil reported that 69% of HPV16 genotypes detected were European variants, reflecting the possible influence of Portuguese—Spanish colonization of the Inca and their descendants of that region[11].

Currently most isolated indigenous groups in the Americas are inhabitants of the Amazonian rainforest, with high rates of cervical cancer and little knowledge of the HPV within these groups. A study by Ong et al.[12] investigated HPV infection in women of three isolated indigenous tribes from the Brazilian Amazon twenty years ago (Tiriyo, Waiapi and Mundurukú). They reported a HPV prevalence of 14.4% but only evaluated 19 types of genital HPV. Genetic sequencing of not typed samples revealed more than 10% nucleotide variability in relation to the nearest genotype, and it was suggested that these isolates were possibly novel types, endemic to native Amazonian population.

Different populations, especially isolated indigenous people, may exhibit distinct characteristics of HPV infections, related to their ancestry, lifestyle and sexual behavior. Moreover, knowledge about the epidemiology of HPV is necessary to understand the etiologic role of different HPV types and variants in these populations in order to develop effective preventative strategies against CC. Isolated indigenous groups (Yanomami) and groups with permanent interaction with the surrounding society (Macuxi and Wapishana) inhabit the extreme north of the Brazilian Amazon, presenting an ecological difference that can be used to investigate the influence of isolation and prehistoric living conditions on HPV diversity and associated cervical disease. To date, the prevalence and diversity of cervical HPV in these groups have not been studied. The objective of this investigation was to evaluate the prevalence of cervical intraepithelial lesions and HPV in indigenous groups from the Amazon region, before the vaccination against HPV.

## Material and Methods

### Ethical Aspects

The study was approved by the IRB of the Tropical Medicine Foundation of the Universidade do Estado do Amazonas. As indigenous peoples are considered vulnerable by the Brazilian government, the study was also reviewed and approved and by the National Committee for Ethics in Research involving Human Beings in Brasília—DF (Document No. 325/2012; Protocol CONEP 16,800). Tribal leaders agreed and formally authorized the execution of the study, but some questions were not approved by them (considered cultural taboo) and were excluded from the questionnaire (menarche, sexual debut, number of sexual partners, previous STD). The Yanomami people have no written language, but only spoken language. So they can not be considered illiterate. Consequently, the study considered the use of fingerprints in the consent form, and this item was fully approved by both IRB. The female participants were widely informed about the study aims and methods, and signed (or fingerprinted) the consent form. Assistance from professional translators was used as necessary. All samples were coded and did not have individual identifying information.

### Study Design

A cross-sectional study was performed, which consisted of selecting a random sample of indigenous women in remote areas of the northern Amazon region of Brazil, to collect biological samples (cervical swabs), between February and July 2013. The study was designed to evaluate the prevalence of cervical intraepithelial lesions by cytology, and HPV infection by PCR amplification and next generation sequencing (NGS) in a pre-HPV vaccination population comparing two groups of women defined below.

### Study site and participants

**Fig 1:**
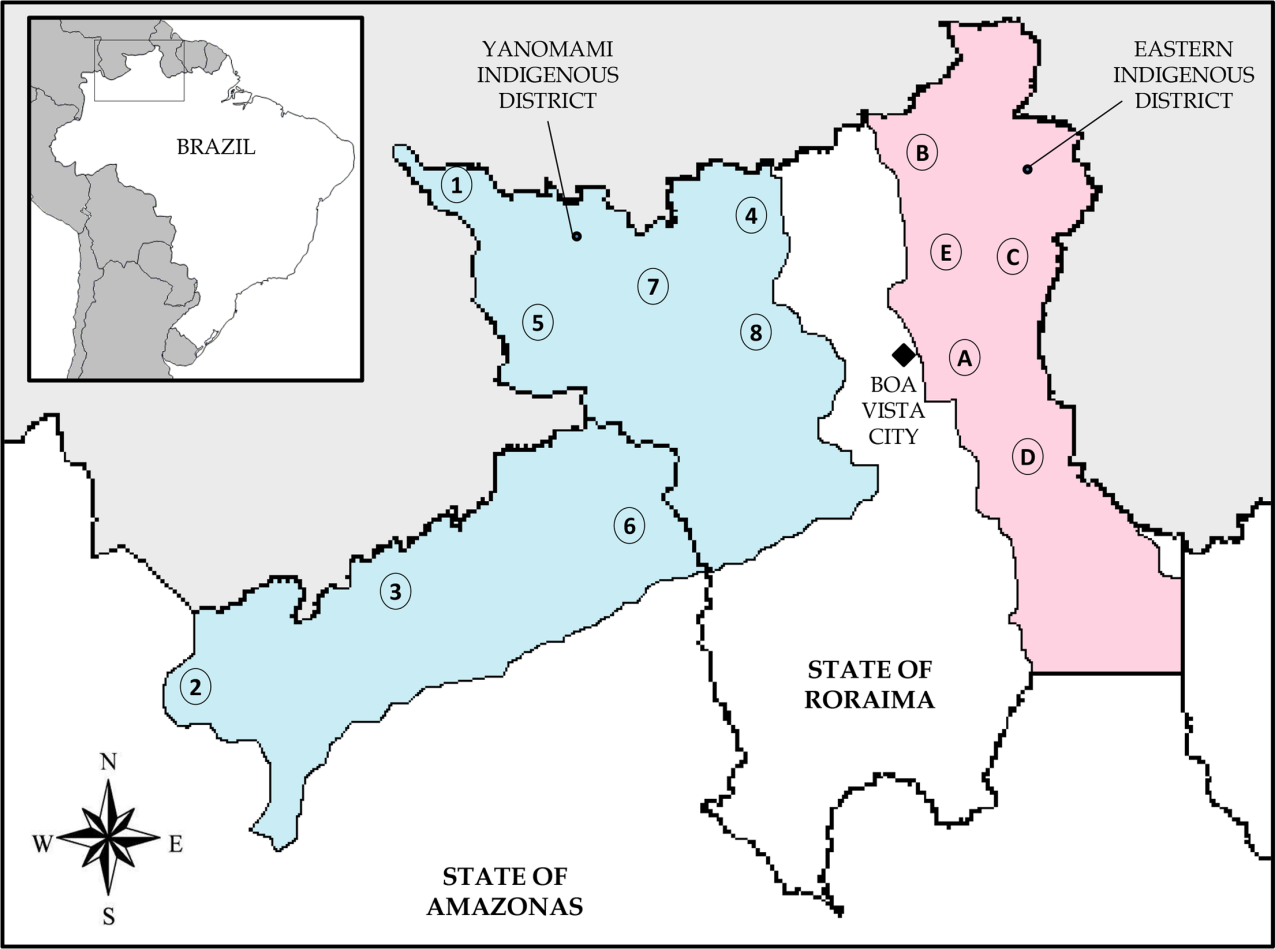
Representative map of the northern Amazonian region of Brazil. Geographical demarcation of the Eastern and Yanomami Indigenous Districts, Brazil, 2014. Location of the randomly selected villages, in order of visit. Yanomami District: 1-Auaris; 2-Maturacá; 3-Marari; 4-Ericó; 5-Surucucu; 6-Demini; 7-Palimi-ú; 8-Alto Mucajaí. Eastern District: A-Malacacheta; B-Camará; C- Vista Alegre; D-Pium; E-Boqueirão. This figure is similar but not identical to the original image, and is therefore for representative purposes only.

Due to cultural, ethnic and anthropomorphic factors, the Federal Government of Brazil has divided the indigenous groups from northern Amazonian region into two geographic regions (i.e., Indigenous Health Districts): the Eastern Indigenous District (composed mainly by Macuxi and Wapishana groups), and the Yanomami Indigenous Disctrict (Yanomami and Yekuana groups) (Fig 1).

With an estimated population of 40,000 inhabitants[4], indigenous people from the Eastern Indigenou District are mainly represented by Macuxi and Wapishana. They belong to the *Karib* linguistic affiliation and the *Pemon* ethnic group. These groups are characterized by having very tenuous ethnic borders with non-indigenous society[13]. The initial contact of Macuxi with western civilization occured during the Portuguese colonial occupation in the mid eighteenth century. Today most members of these ethnic groups are classified as having recent or permanent interactions with the non-indigenous society[14]. They are usually fluent in Portuguese language, and partially adhering to Western culture, life styles and beliefs. Most dwellings allow privacy and usually include running water and plumbing.

Also located in the northern Amazon, in the border region between Brazil and Venezuela, the Yanomami people are made up of approximately 20,000 inhabitants in Brazil[4]. The Yanomami belong to the *Yanomamö* ethnic unity, and have their own linguistic affiliation: *Yanomamam*[15]. They are a typical nomadic society of hunters, farmers and gatherers of the Amazon rainforest, and their contact with non-indigenous people is only very recent in their history[16, 17]. The Yanomami have remained geographically isolated until the early twentieth century. In the decades between 1910 and 1940 the first contact with non-indigenous people was recorded, mainly with military soldiers and anthropologists. Even today, the Yanomami communities are characterized by living in small, isolated groups in the rainforest (accessable only by air), keeping their customs, native languages, social structure and practices such as infanticide, early sexual activity (debut) and multiparity[18]. They live in community thatched huts with dirt floors and neither running water nor plumbing. Geneticists and anthropologists studying the Yanomami have concluded that the absence of genetic, anthropometric and linguistic similarity with indigenous people from the Eastern District supports the view that the Yanomami have been isolated since ancient times[15, 19–22].

### Random sampling

The sample size needed to detect an adjusted Odds Ratio (aOR) higher than 1.5 (2-sided alpha = 0.05) between groups was calculated estimating the prevalence of HPV infection at 20%, based on the results of studies in other indigenous Amazonian women[9, 12]. Considering a confidence interval of 95% and an acceptable error of 5%, a minimum sample size of 600 women was surveyed. As indigenous people are distributed in small villages spread in the Amazon forest with difficult access, the sampling process consisted of random cluster sampling, considering each registered village as a cluster. Forty-two villages in the Yanomami District and 34 in the Eastern District have been currently registered by Brazilian Government. They were numbered, and a random sequence was generated (http://www.random.org/) weighted by the size of the villages. The random selected villages were visited in order of randomization until the target sample size was achieved.

The access to the Eastern District was made by land or waterway, and the access to the Yanomami District was by air (small plane and/or helicopter). Including both groups, 664 women were enrolled. Three women were excluded from the study who either declined sample collection (n = 1) or did not have signs of prior sexual activity during the gynecological examination (n = 2). Among the 661 women included in the study, 18 did not have cytology specimens obtained due to excessive menstrual flow (n = 15) or absence of a cervix and/or dry vaginal swabs (n = 3). Among 643 cervical samples collected, 607 (93.5%) were considered technically satisfactory for cytologic evaluation. The major causes of unsatisfactory samples were desiccation artefacts (n = 13, 2.0%) and/or presence of more than 75% inflammatory cells (n = 20; 3.1%).

### Specimen collection and DNA extraction

Two visits to each indigenous village were necessary. The first to obtain the consent of tribal leaders by explaining the purpose of the study, and to visit families of the village, in order to invite women to participate. During the second visit, interviews, gynecological examination and cervical sample collections were performed in basic health units maintained by the Federal Government of Brazil, located strategically near indigenous villages in remote areas, after formal informed consent of volunteer women, translated to their native language, was obtained. Interpreters speaking the native dialects were used throughout the study. Sexually active women who responded to the invitation were enrolled including women of all ages, pregnant women and postpartum women. Women without prior sexual activity, and those who could not understand the purposes of the study were excluded.

A structured individual interview collected demographic and clinical information, after which the participants underwent a gynecological examination including speculum examination with collection of cervical cells by a phisician, using a cervical brush and wooden spatula, according to the recommendations of the World Health Organization (WHO)[23]. The cells were applied to slides, fixed in the field and analyzed by two senior pathologists. A second cervical sample was collected into specimen transport medium (STM; Qiagen, CA) and DNA was isolated using QIAamp DNA kits (Qiagen, CA) following the recommended protocol. All samples were eluted in an equal volume of TE buffer. All samples were processed in a BioSafety Cabinet in a laboratory physically separated from where the PCR amplification was performed.

### NGS primer design and sample sources

Since the spectrum of HPV types present in this isolated population was unknown, we utilized a novel next-gen sequencing assay recently developed in the Burk laboratory (manuscript in preparation). Briefly, two targeted regions were used for HPV genotyping (see S1 Fig). For each assay, several degenerate primers specific to different HPV genera, species or types were pooled and a unique 8-bp Hamming DNA barcode was appended to the 5’ terminal end of each primer. Each barcode was at least 2-bp different from all other primers[24].

#### Next-Gen PCR amplification and sequencing

An aliquot of each DNA was amplified using 8-bp barcoded oligonucleotide primers for each NGS assay. For all samples, a unique barcode was introduced to the PCR amplicon by the forward and reverse primers. In brief, 1μl DNA sample in a 25μl reaction with an equal amount of AmpliTaq Gold DNA Polymerase (Life Technologies, CA) and HotStart-IT FideliTaq DNA Polymerase (Affymetrix, CA). The PCR conditions included an initial 5 min denaturation at 95°C followed by 15 cycles at 95°C for 1 min, 55°C for 1 min, and 68°C for 1 min, 25 cycles at 95°C for 1 min, 60°C for 1 min, and 68°C for 1 min, and a final extension at 68°C for 10 min. The annealing temperatures of the assay fapR were adjusted to 57°C for the first 15 cycles and 62°C for the second 25 cycles.

Successful amplification of the predicted HPV fragment was estimated for each amplicon by relative band intensity in an ethidium gel compared to a control; barcoded PCR products from all samples were pooled adjusting for DNA concentration of the PCR produtcs by adding less for samples with abudant PCR product. If no amplicon was observed by ethidium bromide staining, 20μl of reaction was added into the pooled mixture. The PCR product mixtures were purified with QIAquick Gel Extraction Kit (Qiagen, CA); an aliquot of purified DNA amplicons was prepared using the Illumina TruSeq DNA Sample Preparation Kit and sequenced on an Illumina HiSeq (Illumina Inc., San Diego, CA) at the Albert Einstein Epigenomics Shared Facility using 150bp paired-end reads.

#### Bioinformatics pipeline and taxonomic classification

**Fig 2:**
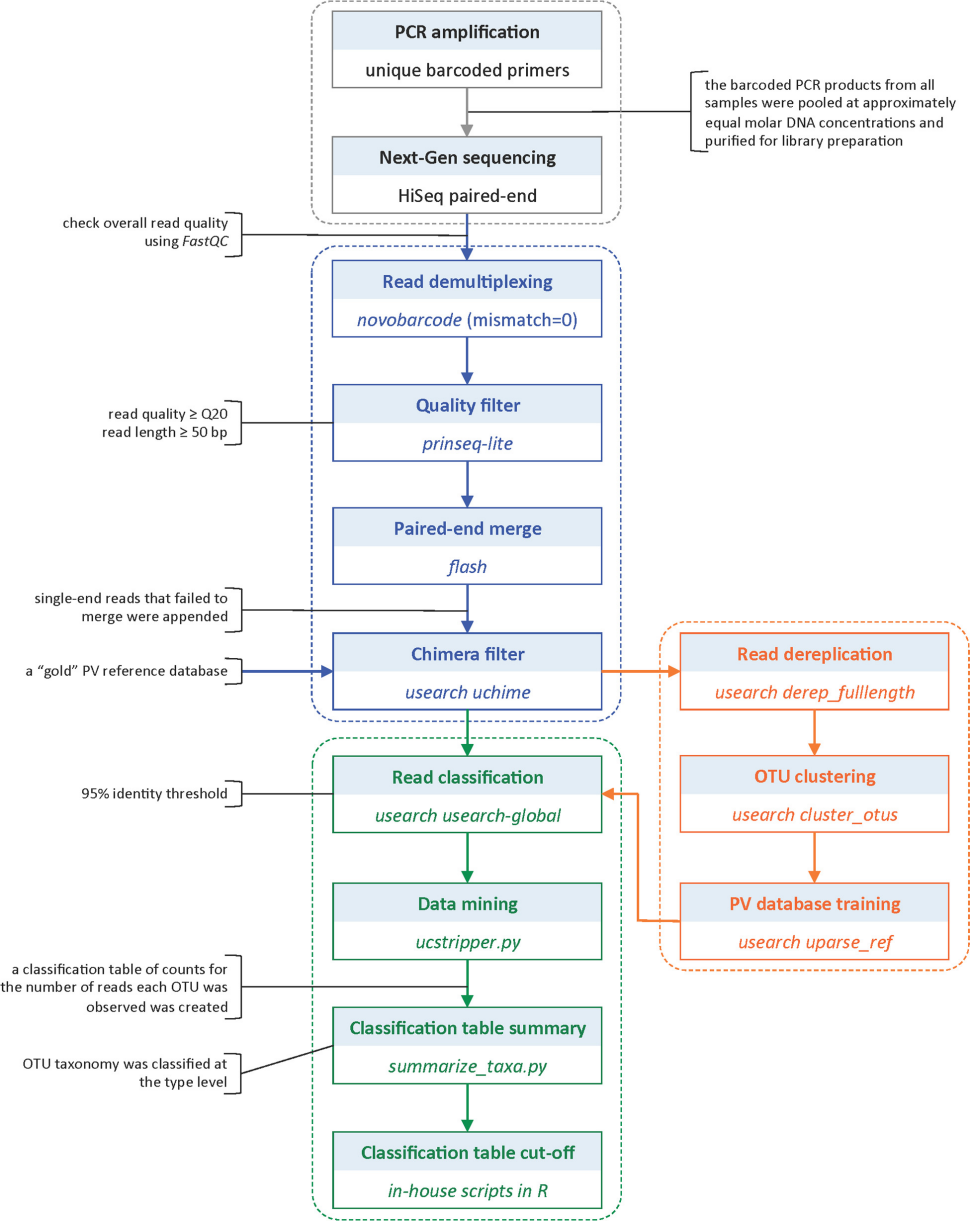
Detailed workflow of bioinformatics pipeline and taxonomic classification of HPV.

The paired-end short Illumina reads were demultiplexed according to their sample-specific barcodes using Novobarcode v1.00 (http://www.novocraft.com/), filtered for low quality reads (Q20) and short length (50 bp) using prinseq-lite v0.20.4 [25], and merged to single reads using FLASh v1.2.7 [26]. The single-end reads that failed to merge were appended. These sequences were then subjected to cull the chimeras using UChime [27] in USEARCH v 7.0.1001 [28] against a “gold” PV reference database. All reads passing the QC filter were then clustered into 95% identity operation taxonomic units (OTUs) and assigned an HPV taxonomy using USEARCH and a training PV dataset. A table of counts for the number of times each HPV type was observed in each sample was created; the taxonomy was classified at the type level (Fig 2).

We created a training PV dataset for taxonomic assignments using the UPARSE algorithm[29]. In brief, all of merged reads were sorted by abundance to discard the singletons in order to improve specificity. The sequence abundances were then OTU clustered within the 95% identify threshold. After chimeric sequences were culled from the data sets, the clustered OTUs were classified using UPARSE-REF algorithm and a PV reference database. Note that the reference sequences were trimmed to only include the region amplified by each NGS assay. If OTUs hitting the reference database had 60%- 90% identities to an exiting PV type, they represented potential novel PV types. A representative OTU set was then assigned with a novel virus (NV) ID and added to the training PV dataset using the most abundant OTU from each bin. The OTUs with less than 60% identities to an exiting PV type were assigned to be non-PV sequences. Several scripts, consisting of a combination of publicly available software and those developed in-house using R v3.0.2, python v2.7.5, perl v5.12.4 and shell to handle the different data and formats were used to assist the reads assignment.

#### OTU cutoff and definition of HPV positivity

Cutoff values were set to remove “low frequency clusters”. Depending on the total number of sequences, we pruned rare OTUs by each NGS assay, each illumina library, or each sequencing lane. For each sample, the taxon (HPV type) was considered negative if the number of reads in the sample was less than 0.5% of the maximum read number of this type in another sample. A sample was considered negative if the total number of HPV reads was less than a defined threshold for each assay. Within each positive sample, for a given HPV type to be considered present, the number of reads had to be greater than 1% of the total number of HPV reads in the sample.

#### Quality control of laboratory procedures

One hundred samples (15%) were randomly selected for repeat amplification and NG sequencing. The assays were performed in a blinded fashion. The concordance of the prevalence of HPV genotypes between the original data and the quality control set was greater than 96% (kappa = 0.90). The following types were considered high-risk HPV (HR-HPV) types: 16, 18, 31, 33, 35, 39, 45, 51, 52, 56, 58, 59, 66[30].

### Methods of data analysis

Standard descriptive statistical analyzes were performed, including frequency distributions for categorical variables, and medians and interquartile ranges (IQR) or means and standard deviations (SD) for continuous variables with non-normal and normal distributions, respectively. Prevalence and 95% confidence intervals (CIs) were estimated based on the binomial distribution. For comparison of sample means (variables with normal distribution and homogeneity of variances) the Student's t test was used. Otherwise, the Mann-Whitney test was used. The χ-square test was used to compare differences in proportions of categorical variables. Odds Ratios (OR) and 95% CIs were calculated in univariate analyzes and adjusted Odds Ratio (aOR) in multivariate logistic regression analyzes. All variables were tested in multivariate analyzes. The ecological diversity of HPV types was calculated using Shannon-Weiner diversity index, and the Mann-Whitney test was used to compare the diversity between groups. Cohen’s kappa coefficient was used to evaluate interobserver agreement. Data were tabulated and analyzed using the software MedCalc version 12.7.0 (Ostend, Belgium).

## Results

In order to study differences between indigenous people representing an ancient life style in the jungle and a more western life style, we selected a random sample from 2 regions of northern Brazil (Fig 1). Thirteen indigenous villages were visited between February and June 2013, and 664 native women were enrolled, representing 76% of the sexually active women within these locals, according to indigenous demographic census (SIASI). Among this sample, 359 (53.9%) women were Macuxi or Wapishana from 5 randomly selected villages in the Eastern District (Malacacheta, Pium-Taiano, Camará, Boqueirão and Vista Alegre), and 305 (46.1%) women were from 8 Yanomami villages (Demini, Maturacá, Marari, Palimi-ú, Surucucu, Alto Mucajaí, Awaris and Ericó). The study was conducted before inclusion of the HPV vaccine in the Brazilian immunization program, and therefore, no woman had been vaccinated.

The average age of participants was 35.8 (±14.5) years, and ranged from 12 to 92 years. Nearly one third of the participants had between 3 and 5 children (n = 241, 36.4%) and 48 (7.2%) women were pregnant at the time of examination. Of 46 women without children (6.9%), 27 had been pregnant at least 1 time. The mean age of the women at first child birth was 18.6 years (±4.1). Most women were married or living with a man (n = 539, 80.9%). Among these, the average age at marriage was 20.6 years (±7.9). Table 1 shows demographic characteristics between the ethnic groups (Table 1). The Yanomami women were younger, married at an earlier age, had a first childbirth at a younger age and were more likely to be currently pregnant.

**Table 1:** Demographic and clinical features of native indigenous women from the northern Brazilian Amazonian region. (Brazil, 2013).

	Total	Yanomami District	Eastern District	p value
**Sample size (n)**	661	305 (46.1%)	359 (53.9%)	**-**
**Age (years)**	35.8 (±14.5)	33.1 (±13.1)	38,1 (±14.6)	0,001
**Number of children**	4.2 (±2.5)	3.5 (±2.3)	4.6 (±2.9)	Ns
**Number of previous pregnancies**	5.1 (±3.0)	4.5 (±2.8)	5.5 (±3.4)	Ns
**Currently pregnant**	48 (7.2%)	32 (10.4%)	16 (4.4%)	0.004
**Age by the birthdate of first child (years)**	18.6 (±4.1)	16.0 (±3.1)	20.1 (±4.8)	0.004
**Marital status**				
Single	91 (13.7%)	34 (11.1%)	57 (15.8%)	Ns
Married/living together	539 (81.5%)	249 (81.6%)	290 (80.3%)	Ns
Widow/divorced	34 (5.1%)	20 (6.5%)	14 (3.9%)	Ns
**Age at the marriage (years) (n = 573)**	19.5 (±5.8)	17.1 (±5.1)	21.5 (±8.3)	0.001
**Had undergone at least 1 Pap test within last 3 years (n = 631)**	366 (58.0%)	118 (42.0%)	248 (70.6%)	<0.0001
**Had undergone at least 1 Pap test life long (>24 years old) (n = 517)**	427 (82.5%)	158 (72.8%)	269 (89.6%)	<0.0001
**Had undergone at least 3 Pap test life long (>24 years old) (n = 517)**	264 (51.0%)	75 (34.5%)	189 (63.0%)	<0.0001
**Previous hysterectomy**	10 (1.5%)	2 (0.6%)	8 (2.2%)	ns

Ns: not significative.

When asked if they had at least one Pap test within the last three years, 366 (58%) of 631 women reported having had a Pap test. Amongst the women aged 25 years or older (n = 517), 427 (82.6%) women reported having been screened for cervical cancer one or more times with 264 (51.0%) women stating they had 3 or more Pap tests. Only 22 (3.3%) women were aware of the results of their prior cytology, and all were from the Eastern District. Eight women from the Eastern District (2.2%) had undergone prior hysterectomy of which 2 were for cervical cancer. Only 2 Yanomami women had undergone hysterectomy and were unaware of the reason for surgery.

Among the 607 cytologic samples considered technically satisfactory, the prevalence of cytologic abnormalities was 3.3% (95% CI: 2.1 to 5.1%). The abnormal smears included 10 cases of ASC-US (1.6%), 7 cases of LSIL (1.15%), 2 cases of HSIL (0.33%) and 1 case of invasive carcinoma (0.17%). The prevalence of abnormal cytology was higher in indigenous women from the Yanomami District than from the Eastern District (5.1% vs 1.8%, respectively, p = 0.04). There was no significant difference in inflammatory infiltrates in the specimens obtained from the Yanomami and Eastern groups (68.0% vs 74.7%, respectively). Table 2 shows cytology and HPV results for each group. The participants diagnosed with HSIL and invasive carcinoma were transferred to the city and subjected to colposcopy and biopsy. The diagnoses were confirmed by histopathology. The patient with invasive cancer was treated with radical hysterectomy (stage Ib FIGO).

**Table 2:** Cervical cell cytology and HPV results.

	Yanomami District	Eastern District	p value
**Satisfactory cytologic samples**	275 (94.2%)	332 (94.5%)	ns
**Any intraepithelial lesion**	14 (5.1%)	6 (1.8%)	0.04
ASC-US	7 (2.5%)	3 (0.9%)	Ns
LSIL	4 (1.5%)	3 (0.9%)	Ns
HSIL	2 (0.7%)	0	Ns
Invasive cancer	1 (0.4%)	0	Ns
**HPV positive (all ages)**	140 (45.9%)	124 (34.5%)	0.003
≤35 years old	88 (44.6%)	79 (44.1%)	Ns
>35 years old	52 (49.0%)	45 (25.0%)	<0.0001
**High-Risk HPV positive (all ages)** ^**&**^	104 (34.1%)	69 (19.2%)	<0.0001
≤35 years old	65 (32.9%)	50 (27.9%)	Ns
>35 years old	39 (36.8%)	19 (10.5%)	<0.0001
**HPV16 positive (all ages)**	29 (9.5%)	10 (2.8%)	0.001
≤35 years old	17 (8.6%)	7 (3.9%)	Ns
>35 years old	10 (9.4%)	3 (1.6%)	0.005
**HPV18 positive (all ages)**	22 (7.2%)	7 (1.9%)	0.003
≤35 years old	14 (7.1%)	6 (3.5%)	Ns
>35 years old	7 (6.6%)	1 (0.5%)	0.008
**HPV multiple type positive (all ages)**	72 (23.6%)	47 (13.0%)	<0.0001
≤35 years old	49 (24.8%)	33 (18.4%)	0.02
>35 years old	23 (21.3%)	14 (7.7%)	<0.0001
**Cumulative HPV types detected/woman** (positive samples)	2.1	1.8	0.02
HPV types detected*	16, 31, 18, 53, 62, 66, 58, 6, 90, 30, 59, 52, 81, 45, 68, 71, 39, 34, 102, 51, 56, 42, 61, 84, 86, 87, 67, 101, 73, 40, 43, 13, 74, 54, 89, 70, 85, 33, 35, 194, 103	31, 68, 53, 66, 6, 62, 59, 16, 58, 52, 30, 18, 81, 74, 71, 34, 54, 86, 89, 39, 101, 61, 87, 56, 45, 40, 67, 42, 90, 171, 84, 91, 33, 11, 44, 72, 26, 69, 70, 85, 35, 79, 51, 103, 108	-
**Diversity of HPV types in positive woman**			
Number of HPV types	42	52	-
Ecological diversity index^#^	0.52	0.39	0.012

^&^High-risk HPV types: 16, 18, 31, 33, 35, 39, 45, 51, 52, 56, 58, 59, 66.

^#^Shannon-Wiener index (natural logarithms).

*In decreasing order of prevalence.

Ns: not significative; ASC-US: atypical squamous cell of unknown significance; LSIL: low grade squamous cell intraepithelial lesion; HSIL: high grade squamous cell intraepithelial lesion.

The overall prevalence of HPV was 39.7% (95% CI: 37.6% to 41.8%). HPV infection was more common in Yanomami women than in Macuxi and Wapishana women (45.9% vs 34.5%, respectively, p = 0.003). Among the positive cases, HPV multiples type infections were detected in 45.1%, and ranged from 2 types (n = 58) to 11 types of HPV (n = 1), with a mean of 1.9 HPV types per positive participant. The prevalence of multiple type infections was higher in women from the Yanomami District than from the Eastern District (45.9% vs 34.5%, respectively; p = 0.02).

**Fig 3:**
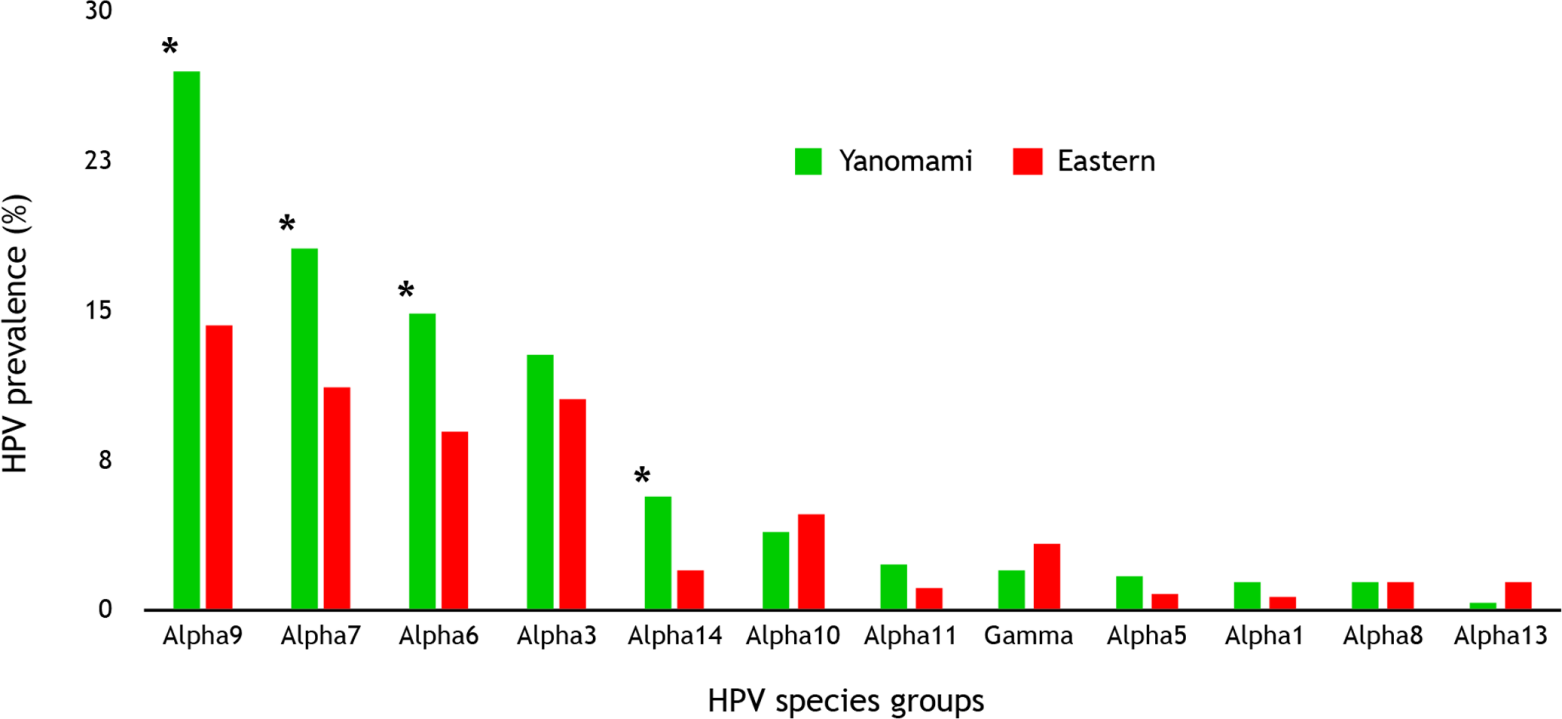
HPV prevalence by HPV species groups. *significative difference of prevalence of HPV between ethnic groups (p<0.05).

**Fig 4:**
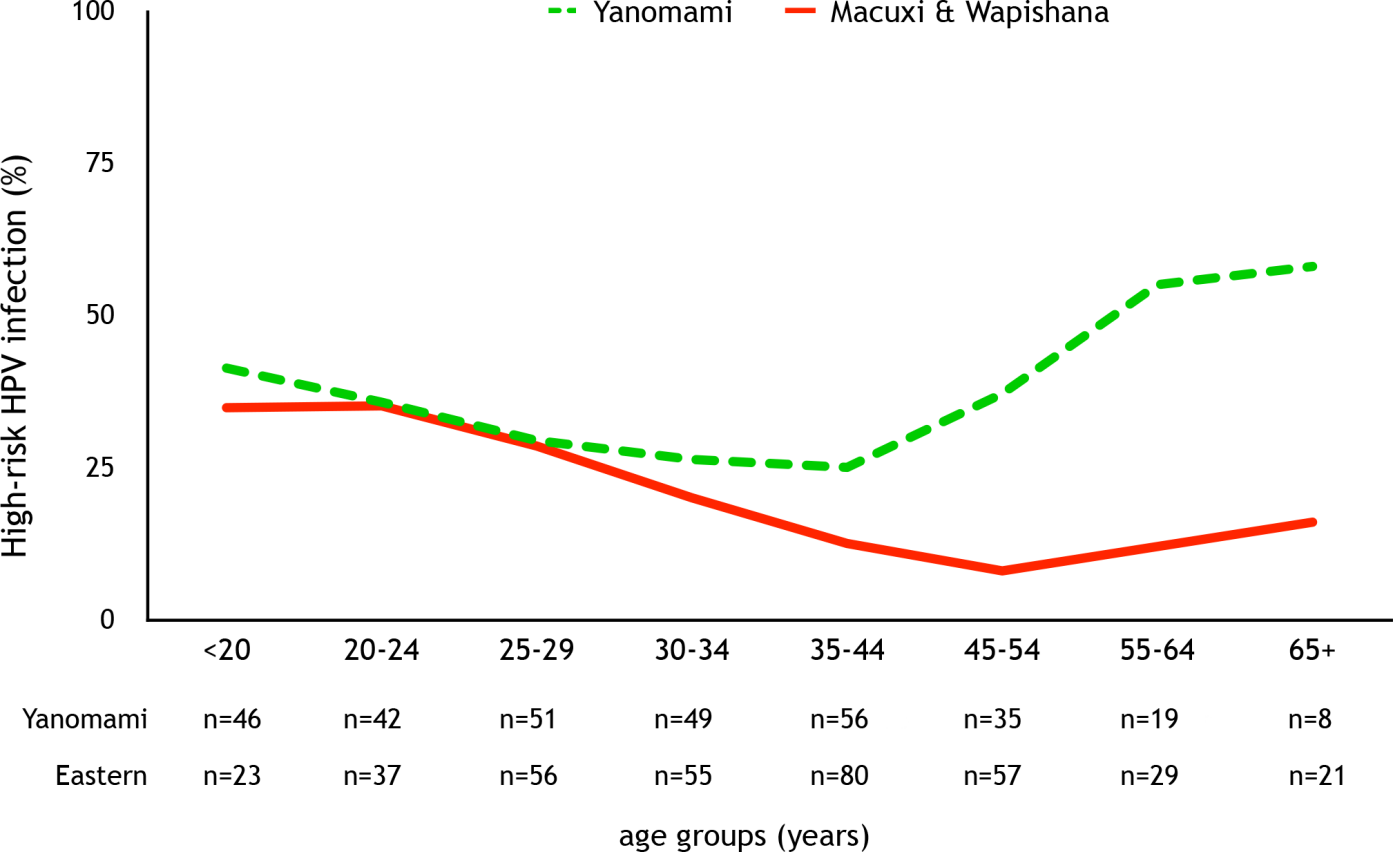
Prevalence of high-risk HPV by age group.

Sixty different HPV types (representing 12 HPV species groups) were detected (Fig 3). In the Yanomami women, 42 HPV types were detected, compared to 52 types in the Macuxi and Wapishana women (Table 2). In addition, analysis of HPV type diversity using the Shannon-Weiner diversity measure indicated there was greater HPV type diversity amongst the Yanomami women. Eight HPV types had sequence variability higher than 5% in relation to the closest genotype in the amplified fragment, and were considered possible novel HPV types. The majority (n = 7; 87.5%) were from the Eastern group. These samples were sequenced twice using the NGS technique. The most prevalent types in Yanomami women were were HPV 16 (n = 29; 9.5%), HPV 31 (n = 27; 8.8%) and HPV 18 (n = 22; 7.2%). Whereas, HPV 31 (n = 17; 8.6%), HPV 68 (n = 14; 3.8%), HPV 53 (n = 11; 3.0%) were the most common in women from the Eastern District. The prevalence of HR-HPV types was 26.5% (n = 173), and was higher in Yanomami women (34.1% vs 19.2%, p <0.0001). The Yanomami women also had a higher prevalence of HPV16 (9.5% vs 2.8%, p = 0.001) and HPV18 infections (7.2% vs 1.9%, p = 0.003) compared to Macuxi and Wapishana women. There was no difference in the prevalence of HPV (or high-risk HPV) between the ethnic groups for participants below 35 years of age (Fig 4). However, considering women older than 35 years, the Yanomami group had a higher prevalence of HPV infection, HR-HPV, HPV16 and HPV18 in relation to the Eastern group (Table 2).

Three cases of HSIL and/or carcinoma were identified in Yanomami women over 45 years infected with HR-HPV types (HPV 16, 16 and 31). The prevalence of HPV infection in the cases of ASC-US and LSIL (n = 17) was 88%, and the most common types were HPV31 (N = 7; 41.1%), HPV16 (n = 3; 17.3%) and HPV 18 (n = 2; 11,7%).

Considering an abnormal cytological test (ASC-US, LSIL and HSIL), univariate analysis revealed that belonging to the Yanomami group increased the chance of atypia (OR = 2.64; IC95% = 1.5–7.1). Age younger than 35 years and/or pregnancy also significantly increased the chance of cytologic abnormality. Ethnicity, age, pregnancy and previous Pap smear tests were evaluated in multivariate analysis. Belonging to the Yanomami ethnic group remained a risk factor, doubling the chance of having altered cervical cytology (aOR = 2.10; 95% CI = 1.1–5.5). Being pregnant also significantly increased the chance of abnormal cytology (aOR = 5.82; IC95% = 2.0–16.6). Age and previous Pap smear were risk factors for cytologic abnormalities in this model. Using HR-HPV infection as an outcome, belonging to the Yanomami group doubled the chance of infection in relation to being Macuxi or Wapishana (OR = 1.98; IC95% = 1.2–2.6). Being single, 35 years old or less, and reporting more than four pregnancies were also significant risk factors. Multivariate analysis of HR-HPV infection identified 2 independent risk factors: being Yanomami increased by 64% the chance of HR-HPV infection (aOR = 1.64; CI 95% = 1.1–2.4), and being single almost doubled the chance of HR-HPV (aOR = 2.22; CI 95% = 1.2–3.8) (Table 3).

**Table 3:** Univariate and multivariate analyses of risk factors for HR-HPV-DNA detection in native indigenous women from northern Amazonian region.

		High-risk HPV infections*			
Demographics and clinical features	Total	Yes (%)	p value	OR (95% CI)	Adjusted OR (95% CI)
Yanomami District	305	104 (34.1%)	<0.0001	2.01 (1.4–2.8)	1.64 (1.1–2.4)
Eastern District	332	69 (19.2%)		1	1
**Pregnant during the test**					
Yes	44	17 (35.4%)	Ns	1.32 (0.7–2.4)	1.24 (0.7–3.1)
No	563	176 (27.2%)		1	1
**Age**					
Less than 35 years old	326	129 (35.2%)	<0.001	1.84 (1.2–2.6)	1.33 (0.8–2.0)
Older than 35 years	279	64 (22.7%)		1	1
**Had undergone at least 1 Pap test life long**					
Yes	471	136 (29.4%)	Ns	0.74 (0.5–1.1)	1.11 (0.4–1.8)
No	136	54 (35.8%)		1	1
**Marital status**					
Single	86	41 (49.9%)	0.0001	2.57 (1.6–4.1)	2.22 (1.2–3.8)
Married–living together	521	136 (27.5%)		1	1
**Had undergone at least 1 Pap test within last 3 years**					
Yes	342	91 (26.8%)	0.01	0.64 (0.4–0.9)	0.77 (0.4–1.3)
No	233	91 (36.4%)		1	1
**Age at the birthdate of 1st child**					
< 20 years old	252	73 (28.9%)	Ns	1.31 (0.3–3.6)	1.11 (0.2–5.4)
≥ 20 years old	141	35 (24.8%)		1	1
**Number of pregnancies**					
>4 pregnancies	286	105 (36.8%)	0.002	1.75 (1.2–2.4)	1.52 (0.8–2.3)
≤4 pregnancies	317	81 (25.0%)		1	1

* High-risk HPV types: 16, 18, 31, 33, 35, 39, 45, 51, 52, 56, 58, 59, 66

Ns: not significative (p<0.05). All variables were adjusted for all other variables in multivariate analysis.

Evaluation of HPV prevalence by village revealed a geographic clustering of regions with a high HPV prevalences. In the Yanomami group, the villages located in the northeast of the Yanomami District (Fig 1) and marked as 4, 7 and 8 presented a higher prevalence of HR-HPV infection than the other dispersed Yanomami villages (50.0% vs 30.3%; p = 0.005; OR = 2.29; CI95% = 1.2–4.0). There was no significant difference in the age (34,2 vs 32,8 years old; respectively; p = 0,8) between the participants from the northeast villages (n = 80) and those from the other Yanomami villages (n = 205). The Yanomami villages located in northeast also presented a higher prevalence of HPV 31, HPV 16 and HPV 66 than other villages. Amongst the Eastern group, the village marked as B (n = 70), located in the north of the Eastern District, had a higher prevalence of HR-HPV infections compared to the other groups (30,0% vs 16,5%; p = 0,01; OR = 2.16; CI95% = 1.1–3.9), and also no significant difference of age between villages were observed.

## Discussion

Significant progress can be observed in recent decades regarding publications on women's reproductive health in the Brazilian context. It is noted however that scientific progress has developed mainly in the urban population[31] [32, 33], remaining an important gap in understanding the determinants of health and disease of indigenous women in Brazil.

This study is the first random sampling of a difficult to access group of women and revealed a high prevalence of HPV in indigenous groups from northern Brazilian Amazonian region, reaching 45.9% in Yanomami women. Despite differences in laboratory methods, this seems to be a higher prevalence than those usually reported in Brazil. Studies on the HPV prevalence are generally concentrated in the more developed regions in the country (south and southeast), ranging from 10.4 to 24.5% in asymptomatic (non-indigenous) women [34–36]. Two studies were performed in Amazon regions. Rocha et al.[37] conducted a population-based study evaluating non-indigenous women of the Amazonian region of Brazil (rural and urban area). They used a Sanger-based sequencing of HPV-DNA, and the prevalence of HPV was 29.1%, considered high by the authors. Similarly to our study, 97% of cytologies were normal. Castro et al.[38] also assessed the HPV prevalence in urban women from the central Amazon region (Brazil). Also using Sanger-based sequencing, they found a HPV prevalence of 25% among asymptomatic women seeking the preventive service, lower than in our study. However it is remarkable that in these studies evaluating urban women of Amazonia, the absolute prevalence of HPV16 was substantially higher than in the indigenous women. Rocha et al.[37] described that almost 60% of HPVs detected were HPV16 (58.1%), an absolute HPV16 prevalence of 16,8%, while Castro et al.[38] reported an absolute HPV16 prevalence of 14,8%. In the Yanomami group, the HPV16 prevalence was 9,5%, and in Makushi and Wapishana group only 2,8%. The opposite was found regarding the HPV18. The HPV18 has not been commonly detected in Brazilian studies[36, 39], or it usually has a prevalence below 1% in asymptomatic women[40, 41]. In the study of Rocha et al.[37] no participant was positive for HPV-18 as well as in Castro’s study[38]. However, HPV18 was the third most common type in our study, present in 7% of Yanomami women (15% of the positive samples), and 2% in the Eastern indigenou group (5% of positive samples).

This study was performed in a non-vaccinated population. A high genotypic diversity of HPV in both indigenous groups was observed, mainly in Yanomami group. While Rocha et al.[37] found 14 types of HPV (16, 33, 81, 6, 70, 31, 35, 45, 52, 53, 61, 68, 71, and 89) and Castro et al.[38] described 7 types of HPV (16, 33, 66, 13, 33, 58, 68) in women from Amazonia, our study detected more than 60 types of HPV. This diversity revealed a high prevalence of non-vaccine HR-HPV, what does not reduce the importance of the vaccination against HPV 16/18 in the indigenous districts, since the majority of non-16/18 HPV infections are cleared by the immune system and do not result in disease. In our study, most cytological alterations were related to types 16, 18 and 31. Furthermore, cross-protection has been described[42] for HPV45 and HPV31. Nevertheless, the development of a nanovalent HPV vaccine recently FDA approved in the USA presents the possibility of better coverage for indigenous women.

The above results classify indigenous women, particularly the Yanomami women, at high risk for HPV infection and cervical cancer. Our study also revealed a significant difference in prevalence of cervical lesions between the two ethnic groups studied, which differed in 2 centuries of interaction with the surrounding society, and that have been coexisting geographically but historically and socially separated. The impact of environmental and socio-cultural changes that take place in virtually all indigenous communities on women's health have pointed to an epidemiologic transition in American indigenous peoples, due to increasing contact with western society and lifestyles[43, 44]. In this context, it was expected that the communities of Eastern District would have a higher prevalence of HR-HPV and precursor lesions of cervical cancer, since they have a longer interaction with the surrounding society in relation to the Yanomami. However, the data consistently point to the opposite. Being a member of the most isolated indigenous group was a risk factor for cervical intraepithelial lesions. This fact, together with the high genotypic diversity, suggests that the Yanomami have sustained HPV infections over a long historical period, preceding the arrival of European settlers in Americas.

Relevant characteristics and cultural practices of reproductive health of Yanomami women were highlighted by Early et al.[17] and may help explain their risk of HPV and CC. This anthropological study reported that Yanomami girls reside in their parent’s house up to 2 years after menarche (12.4 years on average), when they move into the house of the groom's parents and start sexual activity. According to our study, Early reported an average maternal age at the time of first birth of 16 years. In addition to early sexual exposure, they reported a short period between pregnancies, multiparity (fertility rate = 8 children per woman), and multiple sexual partners. The authors also highlighted that during the reproductive period (15 to 40 years), the Yanomami women spend approximately 90% of their time pregnant and/or breastfeeding. An important factor that may contribute to the higher prevalence of cervical intraepithelial lesions among the Yanomami is their geographical isolation in the forest^34^. Difficulties of women’s understanding and acceptance of the screening, infrastructure deficiencies and logistical barriers related to access difficulties may explain the low inclusion of Yanomami women to complete and effective prevention programs. The higher prevalence of Yanomami women who had never had a cytological examination reported in the present study corroborate this notion.

A topic that deserves attention is the relation between HPV and age in these groups. The prevalence of HPV in the Eastern indigenous District presented an L-shaped curve, similar to that described for non-indigenous Brazilian women[41]. However the Yanomami district presented a U-shaped curve. It emphasizes the high and increasing prevalence of HPV and HR-HPV in Yanomami women from 35 years of age, reaching a prevalence of approximately 60% of HR-HPV infection (up to 80% of any type HPV infection) after 55 years. An interesting study[45] described the profile of cervicovaginal cytology (more than 4 million tests) performed in indigenous and non-indigenous women throughout Brazil (between 2008 and 2012). When ethnic groups were compared, the prevalence of HSIL was 2–3 times higher in indigenous women than in non-indigenous women in the elderly group (> 64 year of age). The high prevalence of HR-HPV demonstrated in our study may help explain this finding. A similar curve shape has been observed in native women from rural Nigeria and other regions of Sub-Saharan Africa. Clarke et al.[46] performed a population based study on HR-HPV prevalence in African women and, although they had a lower prevalence of HPV than our study, the authors also described an increasing prevalence of HR-HPV infection after 35 years old. The explanation for this high HR-HPV prevalence at older ages remains elusive. Cultural and behavioral issues, leading to continuing sexual exposure at older ages, and a lower ability of viral clearance among older indigenous women may be implicated in this process. The implication of long-term persistence of HPV infection in isolated hunter-gather peoples from Amazonia and Africa suggests that it could be a characteristic of ancient man and that patterns reflecting decreased prevalence with age in western society represents recent change. These features of the natural history of HPV imply viral-host adaptation to the behaviors and characteristics of the host population for continued viral fitness and success as a replicating symbiotic organism.

This study has limitations. First, the cross-sectional design does not allow the use of temporality and causality criterion, since outcome and risk factors were measured synchronously, so bias and reverse causality could not be eliminated. Second, most Yanomami women did not know the Christian calendar, and they informed their age based on lunar cycles, making this data relatively imprecise, mainly for the older participants, requiring caution in this analysis. However, we have analysed age data using the standard IARC age categories, what strengthen the credibility of the results. Third, data from indigenous census were used to adjust the sample size calculation and to calculate the participation rate. Despite being the best data available, these data can be inaccurate, due to the isolation of the Yanomami tribes.

Despite the identification of 8 novel HPV genomes, only 1 was identified in the most isolated ethnic group. These types were rare, and we were unable to identify novel endemic papillomaviruses in the Yanomami women that are supposed to have been socially isolated from non-indigenous populations for at least 12,000 years. It seems more likely that most, if not all, HPV types evolved thousands of years ago in prehuman primates and took genomic forms similar to those existing today. Nevertheless, future studies of the HPV variants in these women should better disclose their origins.

## Conclusion

Indigenous women from the extreme north of the Brazilian Amazon, especially the Yanomami ethnic group, have increased risk of CC. The high prevalence of HR-HPV, in addition to their geographic isolation and subsequent lower adherence to screening (and treatment) of intraepithelial lesions may contribute to this vulnerability. Yanomami women have relatively higher prevalence of HPV16/18/31 compared to Macushi and Wapishana women, the most oncongenic types. An increasing prevalence and persistence of HPV with age was observed in Yanomami women, which may represent a hunter-gatherer pattern. There was a lower relative and absolute prevalence of HPV16 in relation to that described in non-indigenous women from Brazil, and the opposite was observed for HPV18. Despite the identification of 8 potential novel HPV genomes, and we were unable to demonstrate the presence of novel endemic papillomaviruses unique to the Yanomami women.

## Supporting Information

S1 Fig Primer desing for PCR amplification of HPV-DNA and next generation sequencing. (TIFF)

## References

[1] Ferlay J, Shin HR, Bray F, Forman D, Mathers C, Parkin DM. Estimates of worldwide burden of cancer in 2008: GLOBOCAN 2008. Int J Cancer. 2010;127(12):2893–917. Epub 2011/02/26. 10.1002/ijc.25516 . 21351269

[2] Brasil. Ministério da Saúde. Incidência de Câncer no Brasil—Estimativa 2014. Rio de Janeiro [cited 2014]. Available from: http://www.inca.gov.br/estimativa/2014/.

[3] da Fonseca AJ, Ferreira LP, Dalla-Benetta AC, Roldan CN, Ferreira ML. [Epidemiology and economic impact of cervical cancer in Roraima, a Northern state of Brazil: the public health system perspective]. Rev Bras Ginecol Obstet. 2010;32(8):386–92. Epub 2010/12/25. . 21180875 10.1590/s0100-72032010000800005

[4] Brasil. Ministério da Saúde. SIASI: Sistema de Informação da Atenção da Saúde Indígena. Brasília, 2013. Brasilia. [cited 2013.]. Available from: www.saude.gov.br/siasi.

[5] Jemal A, Bray F, Center MM, Ferlay J, Ward E, Forman D. Global cancer statistics. CA: a cancer journal for clinicians. 2011;61(2):69–90. Epub 2011/02/08. 10.3322/caac.20107 . 21296855

[6] WHO. Health of indigenous people. Geneva 2007 [2013 jul 13]. Available from: http://www.who.int/mediacentre/factsheets/fs326/en/.

[7] Coimbra CEA Jr, Santos RV. Saúde, minorias e desigualdade: algumas teias de inter-relações, com ênfase nos povos indígenas no Brasil. Ciência & Saúde Coletiva. 2000;5(1):125–32.

[8] Kightlinger RS, Irvin WP, Archer KJ, Huang NW, Wilson RA, Doran JR, et al Cervical cancer and human papillomavirus in indigenous Guyanese women. Am J Obstet Gynecol. 2010;202(6):626 e1-7. Epub 2010/05/01. 10.1016/j.ajog.2010.03.015 . 20430361

[9] Brito EB, Martins SJ, Menezes RC. Human papillomaviruses in Amerindian women from Brazilian Amazonia. Epidemiol Infect. 2002;128(3):485–9. Epub 2002/07/13. 12113494 10.1017/s0950268802006908 PMC2869846

[10] Tonon SA, Picconi MA, Zinovich JB, Nardari W, Mampaey M, Badano I, et al Human papillomavirus cervical infection in Guarani Indians from the rainforest of Misiones, Argentina. International journal of infectious diseases: IJID: official publication of the International Society for Infectious Diseases. 2004;8(1):13–9. Epub 2003/12/24. . 14690776 10.1016/j.ijid.2003.03.001

[11] Picconi MA, Alonio LV, Sichero L, Mbayed V, Villa LL, Gronda J, et al Human papillomavirus type-16 variants in Quechua aboriginals from Argentina. Journal of medical virology. 2003;69(4):546–52. Epub 2003/02/26. 10.1002/jmv.10343 . 12601763

[12] Ong CK, Bernard HU, Villa LL. Identification of genomic sequences of three novel human papillomavirus sequences in cervical smears of Amazonian Indians. J Infect Dis. 1994;170(5):1086–8. Epub 1994/11/01. . 7963697 10.1093/infdis/170.5.1086

[13] Dangour AD. Cross-sectional changes in anthropometric variables among Wapishana and Patamona Amerindian adults. Hum Biol. 2003;75(2):227–40. Epub 2003/08/29. . 12943160 10.1353/hub.2003.0031

[14] Salzano FM, Jacques SM, Neel JV. Demographic and genetic relationships among Brazilian Wapishana Indians. Ann Hum Biol. 1980;7(2):129–38. Epub 1980/03/01. . 7425539 10.1080/03014468000004151

[15] Spielman RS, Migliazza EC, Neel JV. Regional linguistic and genetic differences among Yanomama indians. Science. 1974;184(137):637–44. Epub 1974/05/10. . 4820848 10.1126/science.184.4137.637

[16] Early J. The Population Dynamics of the Mucajai Yanomama: Elsevier Science; 1990.

[17] Early JD, Peters JF. The Xilixana Yanomami of the Amazon: History, Social Structure, and Population Dynamics: University Press of Florida; 2000.

[18] Gibbons A. Yanomami people threatened. Science. 1991;252(5013):1616 Epub 1991/06/21. . 2047870 10.1126/science.2047870

[19] Rothhammer F, Neel JV, da Rocha F, Sundling GY. The genetic structure of a tribal population, the Yanomama Indians. 8. Dermatoglyphic differences among villages. Am J Hum Genet. 1973;25(2):152–66. Epub 1973/03/01. 4689037 PMC1762519

[20] Sokal RR, Smouse PE, Neel JV. The genetic structure of a tribal population, the Yanomama Indians. XV. Patterns inferred by autocorrelation analysis. Genetics. 1986;114(1):259–87. Epub 1986/09/01. 3770468 10.1093/genetics/114.1.259 PMC1202935

[21] Ward RH, Gershowitz H, Layrisse M, Neel JV. The genetic structure of a tribal population, the Yanomama Indians XI. Gene frequencies for 10 blood groups and the ABH-Le secretor traits in the Yanomama and their neighbors; the uniqueness of the tribe. Am J Hum Genet. 1975;27(1):1–30. Epub 1975/01/01. 50736 PMC1762774

[22] Neel JV, Tanis RJ, Migliazza EC, Spielman RS, Salzano F, Oliver WJ, et al Genetic studies of the Macushi and Wapishana Indians. I. Rare genetic variants and a "private polymorphism' of esterase A. Hum Genet. 1977;36(1):81–107. Epub 1977/04/07. . 870412 10.1007/BF00390440

[23] Brasil. Ministério da Saúde. Instituto Nacional de Câncer. Coordenação Geral de Ações Estratégicas. Divisão de Apoio à Rede de Atenção Oncológica. Diretrizes brasileiras para o rastreamento do câncer do colo de útero. Rio de Janeiro.: INCA.; 2011.

[24] Smith BC, McAndrew T, Chen Z, Harari A, Barris DM, Viswanathan S, et al The cervical microbiome over 7 years and a comparison of methodologies for its characterization. PLoS One. 2012;7(7):e40425 Epub 2012/07/14. 10.1371/journal.pone.0040425 ; PubMed Central PMCID: PMCPmc3392218. 22792313 PMC3392218

[25] Schmieder R, Edwards R. Quality control and preprocessing of metagenomic datasets. Bioinformatics (Oxford, England). 2011;27(6):863–4. Epub 2011/02/01. 10.1093/bioinformatics/btr026 ; PubMed Central PMCID: PMCPmc3051327. 21278185 PMC3051327

[26] Magoc T, Salzberg SL. FLASH: fast length adjustment of short reads to improve genome assemblies. Bioinformatics (Oxford, England). 2011;27(21):2957–63. Epub 2011/09/10. 10.1093/bioinformatics/btr507 ; PubMed Central PMCID: PMCPmc3198573. 21903629 PMC3198573

[27] Edgar RC, Haas BJ, Clemente JC, Quince C, Knight R. UCHIME improves sensitivity and speed of chimera detection. Bioinformatics (Oxford, England). 2011;27(16):2194–200. Epub 2011/06/28. 10.1093/bioinformatics/btr381 ; PubMed Central PMCID: PMCPmc3150044. 21700674 PMC3150044

[28] Edgar RC. Search and clustering orders of magnitude faster than BLAST. Bioinformatics (Oxford, England). 2010;26(19):2460–1. Epub 2010/08/17. 10.1093/bioinformatics/btq461 . 20709691

[29] Edgar RC. UPARSE: highly accurate OTU sequences from microbial amplicon reads. Nature methods. 2013;10(10):996–8. Epub 2013/08/21. 10.1038/nmeth.2604 . 23955772

[30] Cogliano V, Baan R, Straif K, Grosse Y, Secretan B, El Ghissassi F. Carcinogenicity of human papillomaviruses. The Lancet Oncology. 2005;6(4):204 Epub 2005/04/16. . 15830458 10.1016/s1470-2045(05)70086-3

[31] Coimbra CEA Jr, Garnelo L. Questões de saúde reprodutiva da mulher indígena no Brasil Porto Velho, RO.: Centro de Estudos de Saúde do Índio de Rondônia; 2003 [cited 2013 2013 jul 13]. Available from: http://www.cesir.unir.br/pdfs/doc7.pdf.

[32] Cruz ICFd. A sexualidade, a saúde reprodutiva e a violência contra a mulher negra: aspectos de interesse para assistência de enfermagem. Revista da Escola de Enfermagem da USP. 2004;38:448–57. 10.1590/s0080-62342004000400011 15689003

[33] Riscado JLdS, Oliveira MABd, Brito ÂMBBd. Vivenciando o racismo e a violência: um estudo sobre as vulnerabilidades da mulher negra e a busca de prevenção do HIV/aids em comunidades remanescentes de Quilombos, em Alagoas. Saúde e Sociedade. 2010;19:96–108.

[34] Rosa MI, Fachel JM, Rosa DD, Medeiros LR, Igansi CN, Bozzetti MC. Persistence and clearance of human papillomavirus infection: a prospective cohort study. Am J Obstet Gynecol. 2008;199(6):617e1-7. Epub 2008/09/19. 10.1016/j.ajog.2008.06.033 . 18799155

[35] Girianelli VR, Thuler LC, Szklo M, Donato A, Zardo LM, Lozana JA, et al Comparison of human papillomavirus DNA tests, liquid-based cytology and conventional cytology for the early detection of cervix uteri cancer. European journal of cancer prevention: the official journal of the European Cancer Prevention Organisation (ECP). 2006;15(6):504–10. Epub 2006/11/16. 10.1097/01.cej.0000220630.08352.7a . 17106330

[36] Ayres AR, Silva GA. Cervical HPV infection in Brazil: systematic review. Rev Saude Publica. 2010;44(5):963–74. Epub 2010/09/30. . 20877926 10.1590/s0034-89102010000500023

[37] Rocha DAP, Barbosa Filho RAA, de Queiroz FA, dos Santos CMB. High Prevalence and Genotypic Diversity of the Human Papillomavirus in Amazonian Women, Brazil. Infectious diseases in obstetrics and gynecology. 2013;2013. 10.1155/2013/514859 PMC3755431 23997570

[38] Castro MM, Farias IP, Borborema-Santos CM, Correia G, Astolfi-Filho S. Prevalence of human papillomavirus (HPV) type 16 variants and rare HPV types in the central Amazon region. Genetics and molecular research: GMR. 2011;10(1):186–96. Epub 2011/02/23. 10.4238/vol10-1gmr992 . 21341210

[39] Krambeck WM, Cadide RM, Dalmarco EM, de Cordova CM. HPV detection and genotyping as an earlier approach in cervical cancer screening of the female genital tract. Clinical and experimental obstetrics & gynecology. 2008;35(3):175–8. Epub 2008/08/30. . 18754286

[40] Eluf-Neto J, Booth M, Munoz N, Bosch FX, Meijer CJ, Walboomers JM. Human papillomavirus and invasive cervical cancer in Brazil. British journal of cancer. 1994;69(1):114–9. Epub 1994/01/01. . 8286192 10.1038/bjc.1994.18 PMC1968795

[41] WHO. ICO Information Centre on HPV and Cervical Cancer (HPV Information Centre). Summary report on HPV and cervical cancer statistics in Brazil. [cited 2007]. Available from: http://www.who.int/hpvcentre.

[42] Bonanni P, Boccalini S, Bechini A. Efficacy, duration of immunity and cross protection after HPV vaccination: a review of the evidence. Vaccine. 2009;27 Suppl 1:A46–53. Epub 2009/06/02. 10.1016/j.vaccine.2008.10.085 . 19480962

[43] da Rocha AK, Bos AJ, Huttner E, Machado DC. [Prevalence of metabolic syndrome in indigenous people over 40 years of age in Rio Grande do Sul, Brazil]. Rev Panam Salud Publica. 2011;29(1):41–5. Epub 2011/03/11. . 21390418

[44] Oliveira GF, Oliveira TR, Rodrigues FF, Correa LF, Ikejiri AT, Casulari LA. [Prevalence of diabetes mellitus and impaired glucose tolerance in indigenous people from Aldeia Jaguapiru, Brazil]. Rev Panam Salud Publica. 2011;29(5):315–21. Epub 2011/06/29. . 21709935 10.1590/s1020-49892011000500003

[45] Solé Pla MC, FM; Claro IB; Silva MAF; Dias MBK; Bortolon PC. Descriptive Analysis of the Profile of Cytopathologic Cervical Exams Carried out in Indigenous and Non-Indigenous Women in Brazil, 2008–2011. Revista Brasileira de Cancerologia. 2012;58(3):461–9.

[46] Clarke MA, Gage JC, Ajenifuja KO, Wentzensen NA, Adepiti AC, Wacholder S, et al A population-based cross-sectional study of age-specific risk factors for high risk human papillomavirus prevalence in rural Nigeria. Infectious agents and cancer. 2011;6:12 Epub 2011/08/02. 10.1186/1750-9378-6-12 21801395 PMC3162906

