# Supplementary figures and images for: HPV Infection and Cervical Screening in Socially Isolated Indigenous Women Inhabitants of the Amazonian Rainforest
Source: PLoS One. 2015 Jul 24;10(7):e0133635. doi: 10.1371/journal.pone.0133635 (PMC4514624; doi:10.1371/journal.pone.0133635)

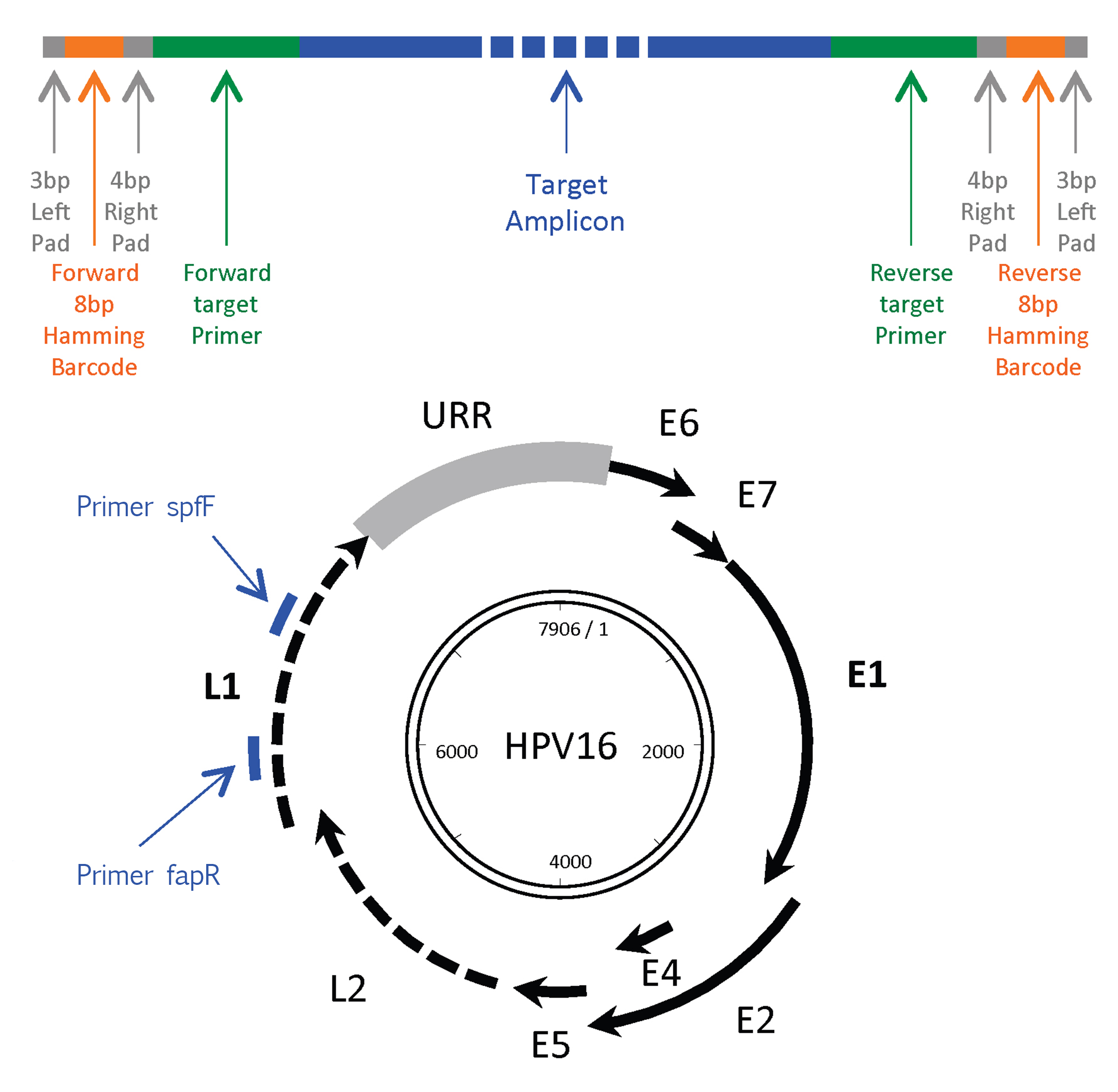

Supplement: S1 Fig — (TIFF) [file pone.0133635.s001.tiff]
